# Flexible Photocatalytic Paper with Cu_2_O and Ag Nanoparticle-Decorated ZnO Nanorods for Visible Light Photodegradation of Organic Dye

**DOI:** 10.1186/s11671-019-3034-7

**Published:** 2019-06-14

**Authors:** Cheng-En Tsai, Shang-Ming Yeh, Chien-Hua Chen, Heh-Nan Lin

**Affiliations:** Department of Materials Science and Engineering, National Tsing Hua University, Hsinchu, 30013 Taiwan

**Keywords:** ZnO nanorod, Cu_2_O nanoparticle, Ag nanoparticle, Photocatalytic paper, Photocatalysis

## Abstract

We report on the fabrication of flexible photocatalytic paper comprised of Cu_2_O and Ag nanoparticle (NP)-decorated ZnO nanorods (NRs) and its application in visible light photodegradation of organic dye. ZnO NRs are first grown on a kraft paper substrate using a hydrothermal method. The NRs are subsequently decorated with Cu_2_O, Ag, or both NPs formed by photoreduction processes. Scanning electron microscopy and X-ray diffraction analysis confirm the crystallinity of ZnO NRs. Transmission electron microscopy analysis confirms the compositions of the two types of NPs. Four different types of photocatalytic papers with a size of 10 × 10 cm^2^ are prepared and used to degrade a 10-μM and 100-mL rhodamine B solution. The paper with Cu_2_O and Ag NP-co-decorated ZnO NRs has the best efficiency with first-order kinetic constants of 0.017 and 0.041 min^−1^ under the illumination of a halogen lamp and direct sunlight, respectively. The performance of the photocatalytic paper compares well with other substrate-supported ZnO nanocomposite photocatalysts. With the advantages of flexibility, light weight, nontoxicity, low cost, and ease of fabrication, the photocatalytic paper has good potential for visible light photocatalysis.

## Introduction

Metal oxide nanomaterials have received extensive research interests in the past two decades for their widespread applications in photonics, electronics, energy, sensing, environmental protection, and so forth [1–6]. Among them, reports on ZnO nanomaterials have been especially abundant due to the ease of growth and morphology control [1, 2]. Photocatalysis using substrate-supported ZnO nanorods (NRs) for photodegradation of organic dye is a potentially important application [2, 7, 8]. ZnO NRs offer the advantages of suitable energy band positions, nontoxic nature, ease of growth, low cost, etc. The use of substrate-supported nanomaterials also avoids a separation process after dye degradation in comparison with the use of dispersed nanomaterials.

The organic dye degradation is due to the strong oxidizing power of hydroxyl radicals, and the generation of hydroxyl radicals is based on the following equations [7, 8]:

1 $$ {\mathrm{OH}}^{-}+{h}^{+}\to {}^{\bullet}\mathrm{O}\mathrm{H} $$

2 $$ {\mathrm{H}}_2\mathrm{O}+{h}^{+}\to {\mathrm{H}}^{+}+{}^{\bullet}\mathrm{O}\mathrm{H} $$

3 $$ {\mathrm{O}}_2+{e}^{-}\to {\mathrm{O}}_2^{\bullet -} $$

4 $$ {\mathrm{O}}_2^{\bullet -}+{\mathrm{H}}^{+}\to {\mathrm{H}\mathrm{O}}_2^{\bullet } $$

5 $$ {\mathrm{O}}_2^{\bullet -}+{\mathrm{H}}^{+}+{\mathrm{H}\mathrm{O}}_2^{\bullet}\to {\mathrm{H}}_2{\mathrm{O}}_2+{\mathrm{O}}_2 $$

6 $$ {\mathrm{H}}_2{\mathrm{O}}_2+{e}^{-}\to {}^{\bullet}\mathrm{O}\mathrm{H}+{\mathrm{O}\mathrm{H}}^{-} $$

As ZnO is a wide band gap semiconductor, a commonly applied approach is to decorate the NRs with narrow band gap semiconductor nanoparticles (NPs) to extend light absorption towards the visible range. Noble metal NPs have also been utilized for the same purpose because of strong visible light absorption caused by the plasmonic effect [9]. Another important concern is that recombination of photogenerated electron-hole pairs is high in ZnO and suppression of recombination is crucial for photocatalysis [7]. The NR and NP heterojunction interface facilitates charge separation and thus enhances photocatalysis. Reports of ZnO NRs decorated with various types of NPs (or alternatively named as nanocomposite) such as ZnSe [10], Ag_2_S [11], CdS [11–13], CuO [14], Cu_2_O [15, 16], ZnFe_2_O_4_ [17], Ag [16, 18, 19], and Au [12, 20] have been abundant in the literature.

For visible light photocatalysis, it seems reasonable to decorate the ZnO NRs with the abovementioned NPs at a large amount to achieve high efficiency. However, excessive decoration of narrow band gap semiconductor or noble metal NPs has adverse effects for the following reasons. Firstly, holes in a narrow band gap semiconductor usually do not have sufficient energy to oxidize hydroxide ions (see Eq. (1)) or water molecules (see Eq. (2)) to hydroxyl radicals. (Reduction potentials of chemical reactions for the formation of hydroxyl radicals will be mentioned later.) Secondly, the plasmonic effect-induced visible absorption of a noble metal NP produces high-energy electrons only but no holes. The generation of hydroxyl radicals due to electrons needs a sequence of chemical reactions (see Eq. (3) to Eq. (6)) and is not as efficient as holes. Thirdly, the presence of defects in ZnO NRs is common [21], and the defects are able to absorb visible light [22]. The photogenerated holes due to defect absorption in ZnO have sufficient energy to generate hydroxyl radicals.

Therefore, simultaneous decoration with both narrow band gap semiconductor and noble metal NPs seems a rational approach to achieve high efficiency. Few works have been reported so far, and indeed, higher photodegradation efficiency has been achieved in comparison with single decoration [12, 16]. The advantages of simultaneous decoration are argued to be enhanced visible light absorption of the semiconductor NPs and faster electron transfer in the ternary nanocomposite, both attributed to the plasmonic effect of noble metal NPs [12].

For practical applications, it is desirable to grow ZnO NRs on flexible substrates. The growth of ZnO NRs on plastic substrates has been well studied [23, 24]. In the past few years, the growth of ZnO NRs on paper substrates starts to attract research attention [25–29] since the paper is flexible, light weight, low cost, environmentally friendly, and easy to handle. However, reports on the growth of NP-decorated ZnO NRs on paper substrates are rare. Furthermore, previously reported works on ZnO NRs grown on paper substrates are mostly related to electronics and sensing [25–28] and very few related to photocatalysis [29]. In the present work, we report on the solution growth of Cu_2_O and Ag NP-decorated ZnO NRs on paper. Good photocatalytic performance of the photocatalytic paper is verified through the degradation of a rhodamine B (RhB) solution.

## Methods

### Growth of ZnO NRs on Paper

ZnO NRs were grown on a kraft paper substrate using the hydrothermal method [1, 2, 7]. Firstly, a ZnO seed solution was prepared using a mixed solution of 10 mM zinc acetate and 3 mM sodium hydroxide (both in ethanol and volume ratio 1:2) heated at 72 °C for 3 h. Secondly, the seed solution was poured on a 10 × 10 cm^2^ kraft paper substrate heated at 90 °C to form a ZnO seed layer. Thirdly, the paper substrate with the ZnO seed layer was immersed in a solution of 25 mM zinc nitrate and 25 mM hexamethylenetetramine (HMTA, C_6_H_12_N_4_) (both in deionized water and volume ratio 1:1) and heated at 95 °C for 7 h in a closed container. The paper substrate was removed from the solution, rinsed several times with deionized water, and dried with nitrogen gas.

### Decoration of Cu_2_O and Ag NPs on ZnO NRs

For surface decoration with Cu_2_O NPs, a NR paper substrate was immersed in a 0.1 mM CuSO_4_ solution and subjected to ultraviolet (UV) irradiation from three 1-W and 254-nm lamps for 1.5 h at 60 °C. For surface decoration with Ag NPs, a NR paper substrate was immersed in a 50-mM AgNO_3_ solution and subjected to UV irradiation for 1 min. The creations of Cu_2_O and Ag NPs are based on the following chemical reactions:

7 $$ \mathrm{ZnO}+ h\nu \to \mathrm{ZnO}+{h}^{+}+{e}^{-} $$

8 $$ {\mathrm{Cu}}^{2+}+2{e}^{-}\to \mathrm{Cu} $$

9 $$ {\mathrm{Cu}}^{2+}+\mathrm{Cu}+{\mathrm{H}}_2\mathrm{O}\to {\mathrm{Cu}}_2\mathrm{O}+2{\mathrm{H}}^{+} $$

10 $$ {\mathrm{Ag}}^{+}+{e}^{-}\to \mathrm{Ag} $$

11 $$ 2{\mathrm{H}}_2\mathrm{O}+4{h}^{+}\to 4{\mathrm{H}}^{+}+{\mathrm{O}}_2 $$

After surface decoration, the paper substrate was rinsed several times with deionized water and dried under nitrogen gas. For decoration with both types of NPs, the Cu_2_O decoration was realized first and then the Ag decoration. Four different types of photocatalytic papers were prepared and designated as ZnO, Cu_2_O/ZnO, Ag/ZnO, and Ag/Cu_2_O/ZnO.

### Photocatalytic Measurement

The photocatalytic activity was evaluated by degrading a 100-mL, 10-μM (~ 4.8 ppm) RhB solution under the illumination of a 300-W halogen lamp. The employed photocatalytic paper was first immersed in an RhB solution in the dark for 1 h, and a new solution was used for photodegradation. A small motor was employed to stir the solution. A 50 μL drop was taken every 10 min during photodegradation. Absorption spectra of the drops collected at different times were measured with the use of an optical microscope equipped with a fiber-connected Si photodiode array spectrometer.

## Results and Discussion

**Fig. 1:**
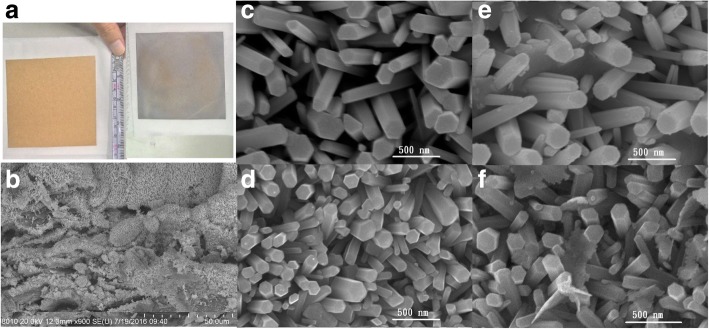
**a** Photographs of a kraft paper substrate (left) and the paper with as-grown ZnO NRs (right). **b** SEM images of the ZnO NR paper. **c**–**f** SEM images of ZnO, Cu_2_O/ZnO, Ag/ZnO, and Ag/Cu_2_O/ZnO NRs, respectively

Figure 1a shows photographs of a 10 × 10 cm^2^ kraft paper and the paper with as-grown ZnO NRs. The brown kraft paper turns gray after ZnO NRs are grown on the paper. The paper has been rolled to form a cylindrical surface (with a radius of around of 2 cm) repeatedly for several times, and no cracks showing the loss of NRs are found when the paper is investigated using an optical microscope. Figure 1b is a scanning electron microscopy (SEM) image of the ZnO paper. Figure 1c is an SEM image of the ZnO NRs. The NRs have a hexagonal shape with diameters ranging between 50 and 300 nm. SEM images of Cu_2_O/ZnO, Ag/ZnO, and Ag/Cu_2_O/ZnO NRs are shown in Fig. 1 d, e, and f, respectively. A small amount of Cu_2_O and Ag NPs can be seen on the NR surfaces.

**Fig. 2:**
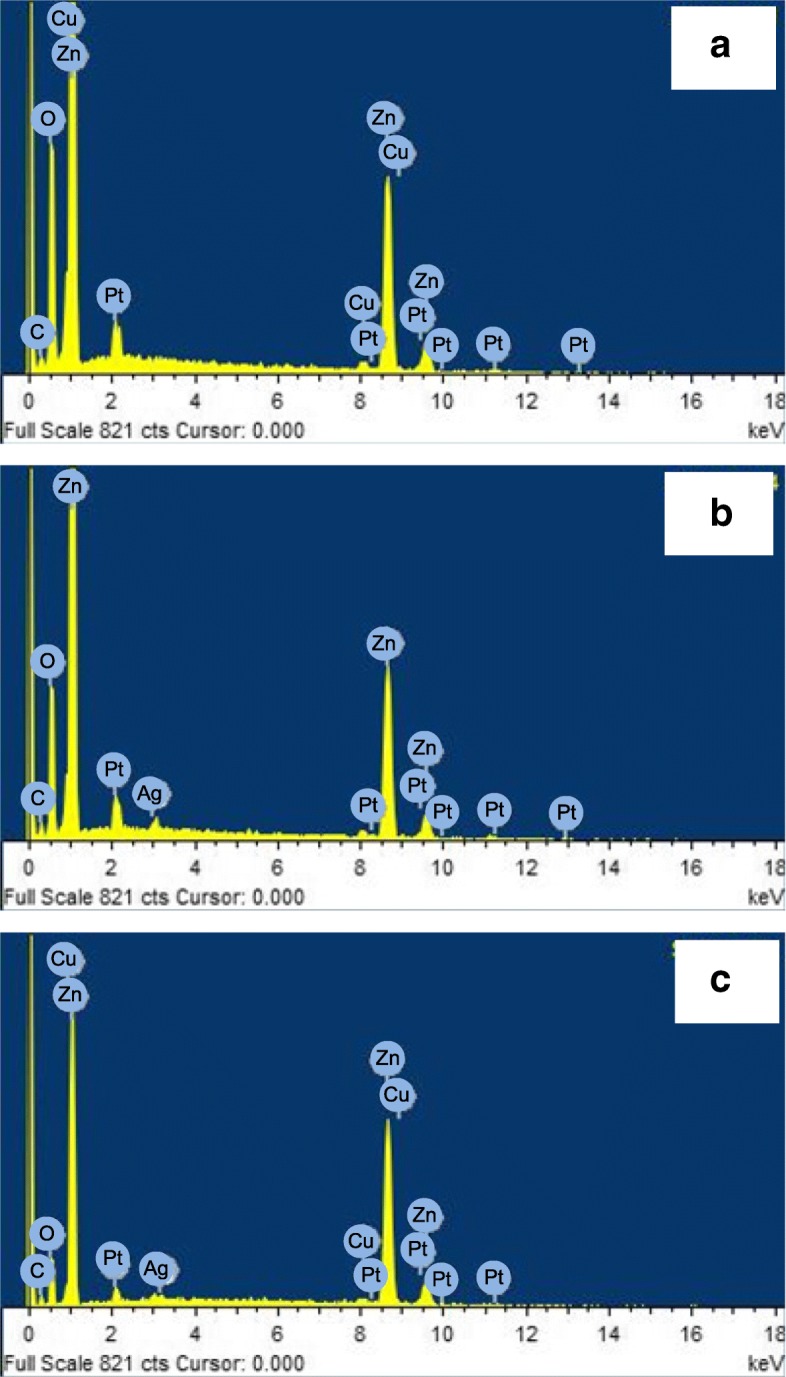
**a–c** EDS spectra of the Cu_2_O/ZnO, Ag/ZnO, and Ag/Cu_2_O/ZnO NRs, respectively

The energy dispersive spectroscopy (EDS) spectra of the Cu_2_O/ZnO, Ag/ZnO, and Ag/Cu_2_O/ZnO NRs are shown in Fig. 2a–c, respectively. The corresponding elements related to the peaks are also indicated. Zn and O peaks are the dominant peaks in the spectra as expected. Pt peaks are from metal coating when taking SEM images. Cu peaks, which are close to the Zn peaks, can be seen in Fig. 2a, indicating the presence of copper oxide. An Ag peak can be seen in Fig. 2b, indicating the presence of silver or silver oxide. Cu and Ag peaks are both found in Fig. 2c, indicating the success of co-decoration.

**Fig. 3:**
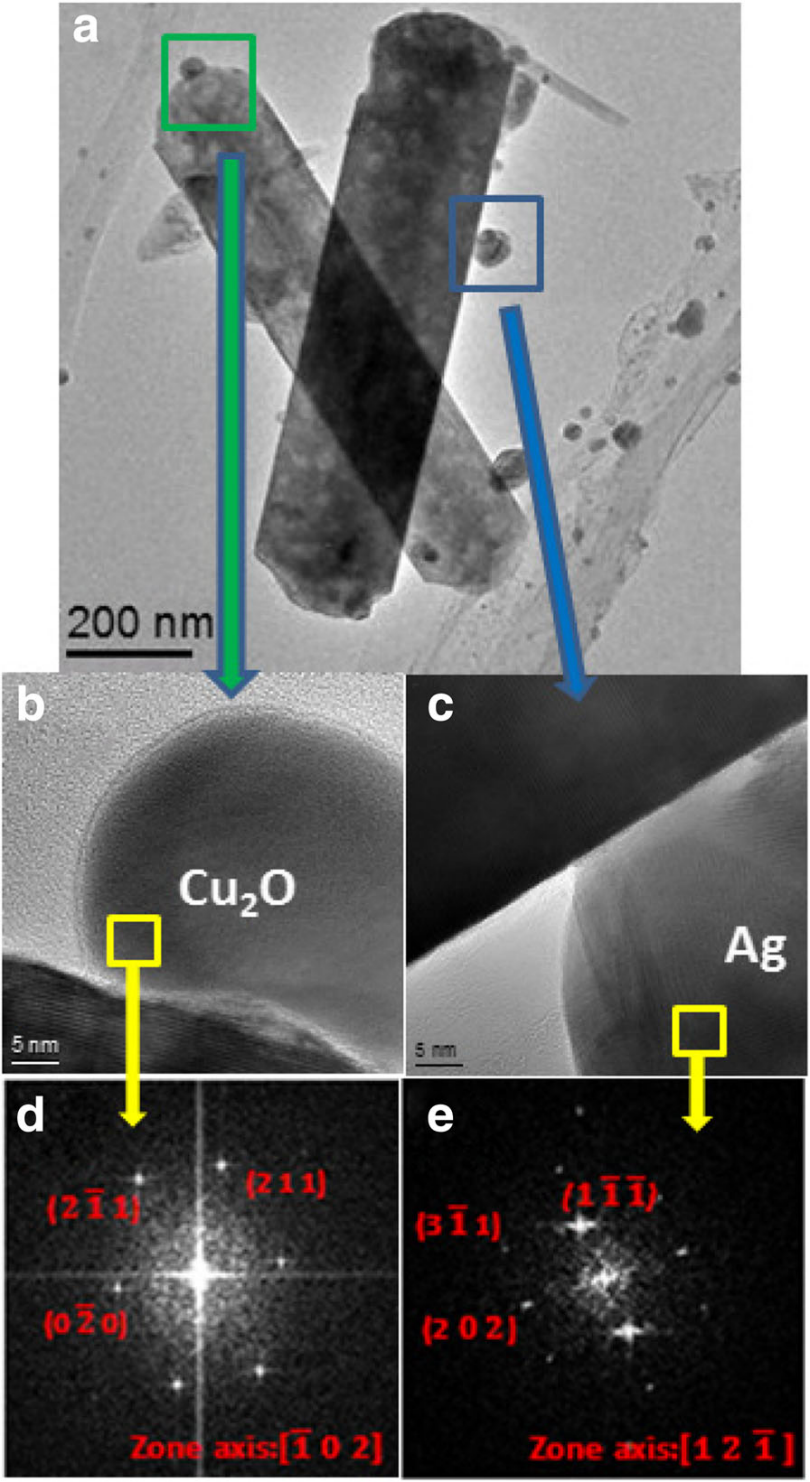
**a** A TEM image of an Ag/Cu_2_O/ZnO NR. **b**, **c** High-resolution TEM images of a Cu_2_O and an Ag NP, respectively. **d**, **e** Fourier transform patterns of the Cu_2_O and the Ag NP, respectively

**Fig. 4:**
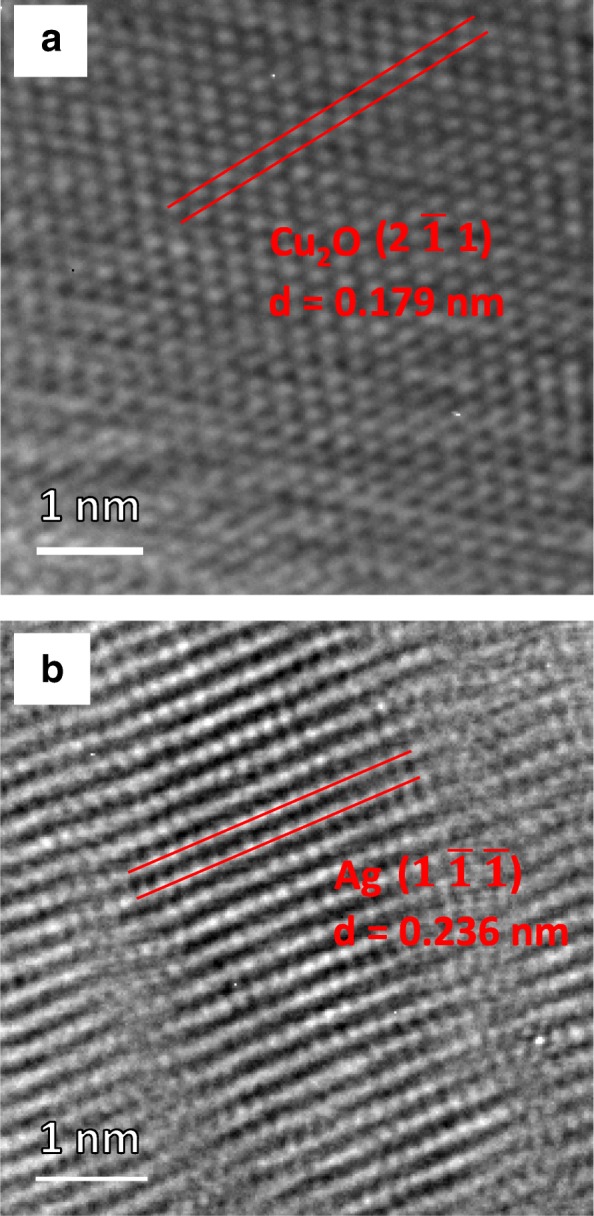
**a**, **b** High-resolution TEM images of a Cu_2_O NP and an Ag NP, respectively

Figure 3a is a transmission electron microscopy (TEM) image of an Ag/Cu_2_O/ZnO NR. High-resolution TEM images of a Cu_2_O and an Ag NP are shown in Fig. 3 b and c, respectively. Fourier transform patterns of the Cu_2_O and the Ag NP are revealed in Fig. 3 d and e, respectively. In Fig. 4a, the $\left(2\kern0.5em \overline{1}\kern0.5em 1\right)$ interplanar spacing is determined to be 0.179 nm, which is consistent with the (2 1 1) spacing of 0.174 nm for Cu_2_O in the Joint Committee on Powder Diffraction Standards (JCPDS) card files. In Fig. 4b, the $\left(1\kern0.5em \overline{1}\;\overline{1}\right)$ interplanar spacing is determined to be 0.236 nm, which is consistent with the (1 1 1) spacing of 0.236 nm for Ag in the JCPDS card files. It is thus confirmed that both types of NPs are formed on the NR surface.

**Fig. 5:**
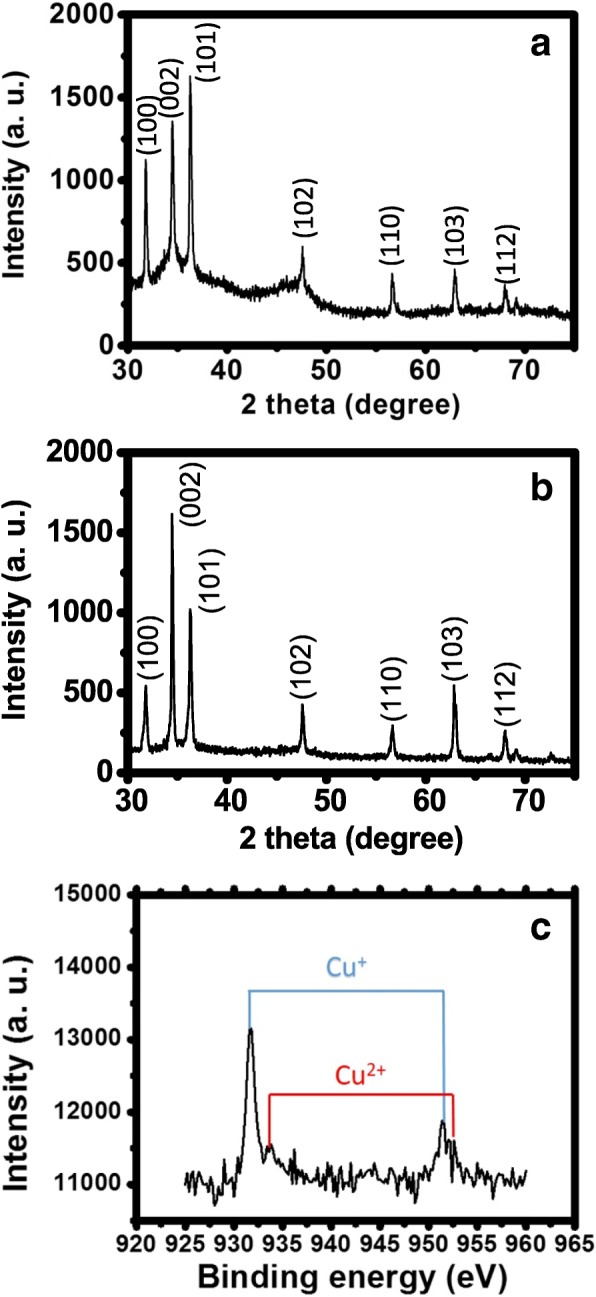
**a**, **b** X-ray diffraction patterns of as-grown ZnO and Cu_2_O/ZnO NRs. **c** An XPS spectrum of Cu_2_O/ZnO NRs

Figure 5a is an X-ray diffraction pattern of as-grown ZnO NRs. The strong peaks coincide with characteristic peaks of ZnO, which confirm good crystallinity of the NRs. Figure 5b is an X-ray diffraction pattern of Cu_2_O/ZnO NRs. It is similar to Fig. 5a, and diffraction peaks of Cu_2_O cannot be observed. This can be reasonably attributed to the small amount of NPs on the NR surface as can be seen in Fig. 3a. Figure 5b also confirms that the crystallinity of ZnO is preserved after the photoreduction process. Figure 5c shows the X-ray photoemission spectroscopy (XPS) of the Cu_2_O/ZnO NRs. The peaks corresponding to Cu^+^ can be clearly seen, which confirms the formation of Cu_2_O NPs. There are also peaks corresponding to Cu^2+^ with smaller intensity, which is most likely due to residual CuSO_4_ or formation of CuO NPs.

**Fig. 6:**
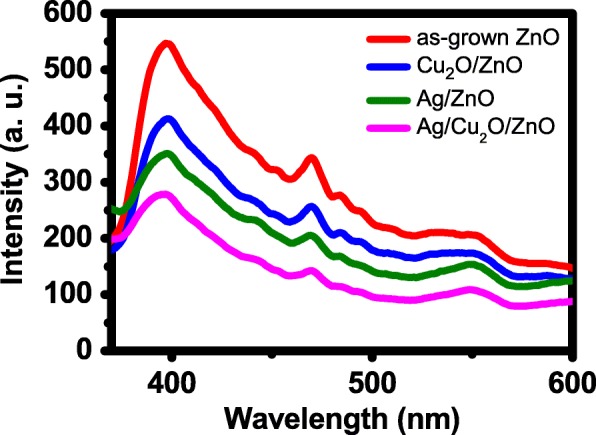
PL spectra of the four photocatalytic papers

Figure 6 shows the photoluminescence (PL) spectra of the four photocatalytic papers. The spectra exhibit a strong peak of band gap emission at around 400 nm and a smaller peak at around 470 nm, which is related to defect emission of oxygen vacancies [22]. The PL intensities of the three NP-decorated ZnO NR papers are less than that of the ZnO NR paper. This is mainly due to two effects. The first is the improved charge separation after the addition of NPs, which reduces the recombination of photogenerated electron and holes. The second is the absorption of the emitted light by the NPs. The Ag/ZnO paper has a lower PL intensity than the Cu_2_O/ZnO paper. The work function of Ag crystal is around 4.5~4.7 eV and a Schottky junction is formed between Ag and ZnO [9]. The junction is effective for charge separation, and as a result, the PL intensity of the Ag/ZnO paper is thus lower. Furthermore, the PL intensity of the Ag/Cu_2_O/ZnO paper is the smallest, which is expected since it has two types of NPs and best charge separation as a result.

**Fig. 7:**
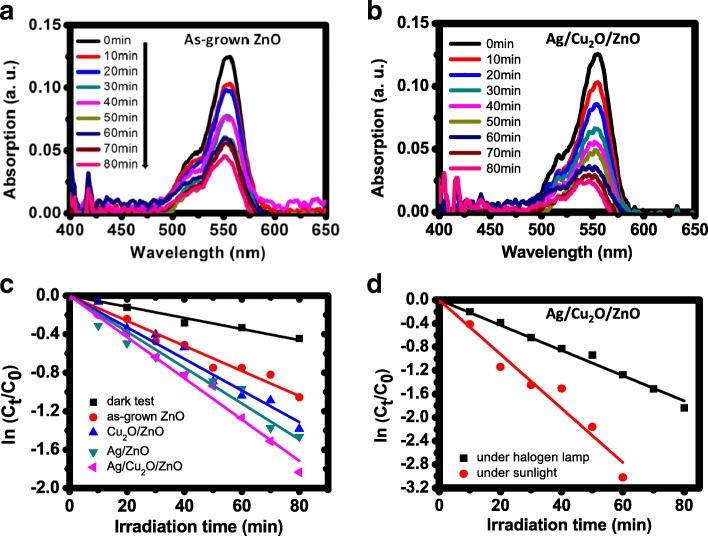
**a**, **b** Absorption spectra of the RhB solution as a function of time (at a 10-min interval) resulting from the photocatalysis of the ZnO and the Ag/Cu_2_O/ZnO papers, respectively. **c** Plots of ln(*C*_*t*_/*C*_0_) for the ZnO paper in the dark and the four photocatalytic papers under the light. **d** Plots of ln(*C*_*t*_/*C*_0_) for the Ag/Cu_2_O/ZnO paper under the illumination of a halogen lamp and direct sunlight

The absorption spectra of the RhB solution as a function of time (at a 10-min interval) resulting from the photocatalysis of the ZnO and Ag/Cu_2_O/ZnO papers under the illumination of a halogen lamp are shown in Fig. 7 a and b, respectively. At 80 min, the residual RhB concentrations are roughly 35% and 16% of the original value, respectively. The ratios of the concentration *C*_*t*_ to the initial concentration *C*_0_ as a function of time for the four photocatalytic papers are plotted in a logarithmic scale in Fig. 7c. The photodegradation results can be fitted to the first-order kinetic equation *C*_*t*_ = *C*_0_ exp(−*kt*), where *t* is the time and *k* the first-order constant. Using least-squares linear fitting, the fitted constants are 0.013, 0.016, 0.019, and 0.022 min^−1^ for ZnO, Cu_2_O/ZnO, Ag/ZnO, and Ag/Cu_2_O/ZnO papers, respectively.

Part of the measured dye degradation is due to dye absorption by the paper. The concentration change caused by the ZnO paper in the dark is also shown in Fig. 7c, and the kinetic constant is 0.005 min^−1^. After subtracting the absorption constant from the measured constants, the corrected photodegradation kinetic constants are 0.008, 0.011, 0.014, and 0.017 min^−1^ for the four photocatalytic papers. Indeed, the Ag/Cu_2_O/ZnO paper shows the best efficiency and the kinetic constant enhancements over the other three papers are around 113%, 55%, and 21%, respectively. The Ag/Cu_2_O/ZnO paper (the same one as was used before) has also been tested under direct sunlight. (The test location was at 120.99° E and 24.79° N. The test date was at noon in July with a temperature of around 32 °C. The light intensity was around AM 1.0.) The results are plotted in Fig. 7d along with the halogen lamp results for comparison. The fitted kinetic constant is 0.041 min^−1^ (after excluding the physical absorption effect), and the value is roughly 2.4 times the kinetic constant obtained by using the halogen lamp.

**Fig. 8:**
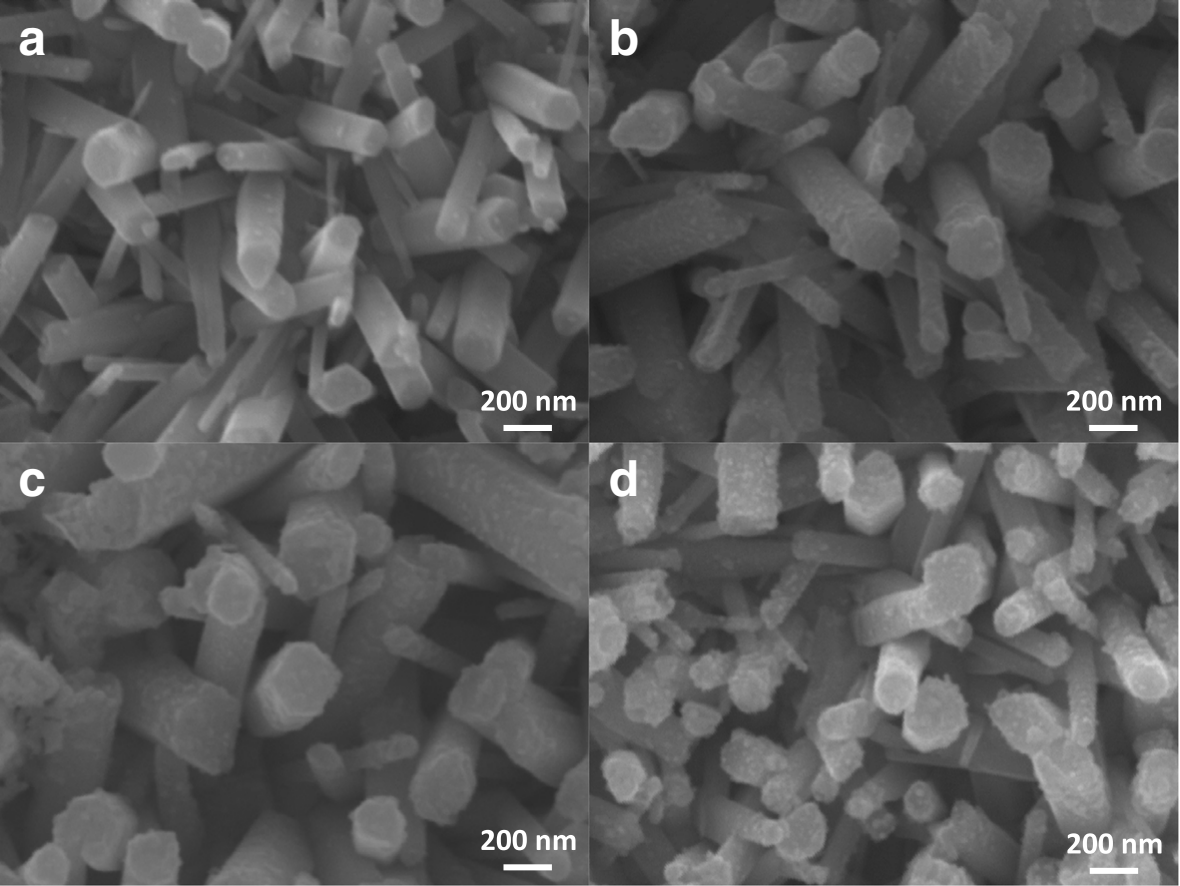
**a–d** SEM images of Ag/ZnO NRs with photoreduction times of 1, 1.5, 2, and 2.5 min, respectively

In the TEM image shown in Fig. 3a, there are only few Cu_2_O and Ag NPs on a ZnO NR. The amount of NPs can be easily increased with the use of longer photoreduction time. The SEM images of Ag/ZnO NRs with different reduction times of 1, 1.5, 2, and 2 min are shown in Fig. 8. In Fig. 8a, there are only some Ag NPs on the ZnO NRs at the reduction time of 1 min. The amount of Ag NPs increases rapidly when the reduction time is slightly increased to 1.5 min as shown in Fig. 8b. Eventually, the ZnO NRs are covered with Ag NPs when the reduction time is increased to 2 min as shown in Fig. 8d. As a result, the photoreduction time can be used as an effective parameter for controlling the amount of Ag NPs.

**Fig. 9:**
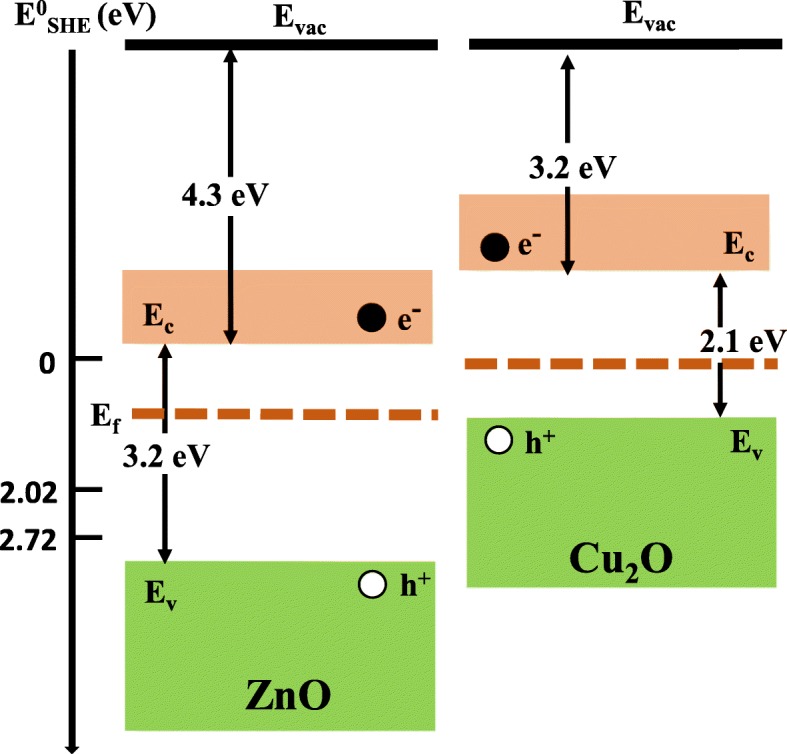
The energy band diagrams of ZnO and Cu_2_O and the standard reduction potentials for Eqs. (1) and (2). The reduction potential is relative to SHE, which is 4.44 eV below the vacuum level

In Fig. 7c, the Ag/ZnO paper shows better efficiency than the Cu_2_O/ZnO paper. This is consistent with the PL result shown in Fig. 6, which indicates the Ag/ZnO paper has lower PL intensity and thus better charge separation. However, it can also be explained by considering the energy band of Cu_2_O. Hydroxyl radicals are responsible for dye degradation and generated from the oxidation of hydroxide ions through Eq. (1) or water molecules through Eq. (2). The energy band diagrams of ZnO and Cu_2_O and the standard reduction potentials for the two reactions are shown in Fig. 9. The work functions of ZnO and Cu_2_O are 4.3 and 3.2 eV, respectively, and the band gap energies are 3.2 and 2.1 eV, respectively. The standard reduction potentials (relative to standard hydrogen electrode (SHE), which is 4.44 V below the vacuum level) are *E*^o^(^•^OH/OH^−^) = 2.02 V and *E*^o^(^•^OH/H_2_O) = 2.72 V, respectively. Therefore, photogenerated holes in Cu_2_O do not have enough energy to participate in the above two reactions. On the other hand, Ag NPs enhance visible light defection absorption in ZnO and the photogenerated holes in ZnO have enough energy to produce hydroxyl radicals. As a result, the Ag/ZnO paper shows better efficiency than the Cu_2_O/ZnO paper.

Table 1 shows a comparison of the performance of the Ag/Cu_2_O/ZnO paper with recently reported substrate-supported NP-decorated ZnO NRs for photodegradation of RhB solution. Although the experimental conditions in these works differ substantially, the reasonable efficiency of the photocatalytic paper is obvious. Considering the advantages of flexibility, light weight, nontoxicity, low cost, and ease of fabrication and scaling up, the present photocatalytic paper has good potential for effective degradation of organic dye pollutants and other photocatalytic applications as well.

**Table 1:** A comparative table of photodegradation of RhB solutions

Material	Substrate	*A* (cm^2^)	*C* (μM)	*V* (mL)	*k* (min^−1^)	Light source	Ref.
CdS/ZnO	Copper fiber	50	~ 21	100	0.033	500-W Xe lamp (UV filtered)	[13]
CuO/ZnO	Glass	4	10	40	0.0076	400-W Hg lamp (UV filtered)	[14]
ZnFe_2_O_4_/ZnO	Sapphire	1	20	5	0.012	150-W Xe lamp (UV filtered)	[17]
Au/ZnO	Si	0.5	5	10	0.031	100-W light bulb	[20]
CdS/Au/ZnO	Steel	4	~ 21	20	0.0039	Solar simulator (UV filtered)	[12]
Ag/Cu_2_O/ZnO	Paper	100	10	100	0.017	300-W halogen lamp	Present work

Catalysts of NP-decorated ZnO NRs or ZnO NR nanocomposites on various types of substrates are used for the photodegradation of RhB solutions. *A*, *C*, and *V* stands for area, concentration, and volume, respectively

## Conclusions

In this study, the fabrication of photocatalytic paper comprised of Cu_2_O and Ag NP-decorated ZnO NRs is reported. The ZnO NRs are grown on a kraft paper substrate using a hydrothermal method, followed by decoration with Cu_2_O, Ag, or both NPs using photoreduction processes. SEM and X-ray diffraction analysis confirm that the NRs possess good crystallinity. TEM analysis confirms the compositions of the two types of NPs. Four different types of photocatalytic papers with a size of 10 × 10 cm^2^ are prepared and used for the photodegradation of RhB solution (10 μM and 100 mL). Under the illumination of a 300-W halogen lamp, the first-order kinetic constants for the ZnO and Ag/Cu_2_O/ZnO papers are 0.008 and 0.017 min^−1^, respectively. Under direct sunlight, the Ag/Cu_2_O/ZnO paper achieves a kinetic constant of 0.041 min^−1^. The performance of the Ag/Cu_2_O/ZnO photocatalytic paper compares well with other substrate-supported ZnO nanocomposite photocatalysts. With the advantages of flexibility, light weight, nontoxicity, low cost, ease of fabrication, and reasonable efficiency, the photocatalytic paper has good potential for reducing organic dye pollution and other visible light photocatalytic applications.

## Data Availability

The datasets used and/or analyzed during the current study are available from the corresponding author on reasonable request.

## Abbreviations

EDS: Energy dispersive spectroscopy
JCPDS: Joint Committee on Powder Diffraction Standards
NP: Nanoparticle
NR: Nanorod
PL: Photoluminescence
RhB: Rhodamine B
SEM: Scanning electron microscopy
SHE: Standard hydrogen electrode
TEM: Transmission electron microscopy
UV: Ultraviolet
XPS: X-ray photoemission spectroscopy
